# Statin therapy and mortality in critically ill heart failure patients: Insights from a triangulated real-world design study

**DOI:** 10.1371/journal.pone.0334822

**Published:** 2025-10-17

**Authors:** Li Wang, Yibing Wang

**Affiliations:** 1 Second Department of Cardiology, The Second Hospital of Hebei Medical University, Shijiazhuang, China; 2 Department of Neurosurgery, Handan Central Hospital, Handan, China; UN Mehta Institute of Cardiology and Research Center, INDIA

## Abstract

This study is aimed to evaluate the association between statin therapy and intermediate-term survival in critically ill patients with heart failure (HF). Using a real-world retrospective cohort from the MIMIC-IV database, we assessed all-cause mortality at 90 and 180 days following ICU admission. To reduce confounding factors, we applied a triangulated analytic framework incorporating propensity score matching, inverse probability of treatment weighting and standardized mortality ratio weighting. Survival outcomes were additionally examined across different statin types, including atorvastatin, rosuvastatin, simvastatin and pitavastatin, using stratified Kaplan–Meier analysis. Among over 8,000 eligible patients, statin use during hospitalization was consistently associated with reduced mortality at both time points across all models. Stratified survival curves showed comparable trends among the different statin types. These findings suggest a potential class-wide survival benefit of statin therapy in the ICU setting for HF patients and highlight the need for further studies to determine whether specific statin selection offers additional clinical advantages.

## Introduction

Globally, Heart failure (HF) continues to contribute substantially to disease burden and death, with the impact being especially pronounced in older adults and those requiring intensive care [1,2]. Despite advances in pharmacological and device-based therapies, outcomes for hospitalized patients with HF remain poor, especially among those requiring intensive care unit (ICU) admission [3,4].

Statins, widely used lipid-lowering agents, exert their primary effect by reducing low-density lipoprotein cholesterol (LDL-C) level. Apart from their cholesterol-lowering action, statins are increasingly recognized for their pleiotropic effects, including modulation of inflammation, oxidative balance and vascular endothelial integrity, potentially contributing to cardio-protective effects during acute decompensated events [5–7]. Multiple studies have indicated that statin medications confer benefits in terms of reducing hospitalization and mortality rates in patients with heart failure [8–11]. However, the role of statins in managing HF remains controversial. Notably, major trials, including CORONA [12] and GISSI-HF [13], did not show significant survival advantages in individuals with chronic forms of the disease, fueling continued debate on their therapeutic value.

Using large-scale real-world data, this study assessed the association between in-hospital statin use and short-term (90-day) and intermediate-term (180-day) all-cause mortality among critically ill patients with HF. We employed a robust statistical framework integrating multiple methodologies, including Cox proportional hazards modeling, propensity score matching (PSM), Inverse probability of treatment weighting (IPTW) and standardized mortality ratio weighting (SMRW). A series of detailed subgroup and interaction analyses was carried out to examine the uniformity of therapeutic outcomes among different clinical strata. Our goal was to generate a more reliable, nuanced and clinically meaningful understanding of statin use in this high-risk population.

## Methods

### Data source and ethics statement

We extracted patient-level data from MIMIC-IV (version 2.2) [14], a mature, open-access critical care database containing de-identified clinical records of ICU admissions at Beth Israel Deaconess Medical Center between 2008 and 2019. Using PostgreSQL queries, we identified patients with heart failure who had or had not been exposed to statins. The MIMIC-IV database has received ethical approval from the Institutional Review Boards (IRBs) of the Massachusetts Institute of Technology and Beth Israel Deaconess Medical Center. Owing to the fully anonymized nature of the data, this retrospective study was exempt from IRB review by the Second Hospital of Hebei Medical University and informed consent was waived. Formal database access was granted to one of the investigators (Yibing Wang, Certification ID: 12722170) under the MIMIC data use agreement. The dataset was accessed on 14 January 2024; all records are fully de-identified and the authors had no access to information that could identify individual participants during or after data collection. All analyses followed the Declaration of Helsinki and the STROBE guidelines [15].

### Study population

Adult patients aged 18 years and above who had been hospitalized with a diagnosis of HF were included in this study. The diagnosis of HF was determined using relevant ICD-9 and ICD-10 classification codes. To avoid duplication and ensure independence of observations, only the first hospitalization record was retained for individuals with multiple admissions. Patients with a hospitalization duration of less than 72 hours or with incomplete data were excluded.

### Drug exposure

The primary exposure variable was statin use during hospitalization, defined as receiving at least one dose of any statin (such as atorvastatin, rosuvastatin and simvastatin). Statin use was identified from prescription and administration records in the prescriptions and inputevents tables of the database. Patients were classified into the “statin group” and the “non-statin group” according to statin exposure.

#### Statin intensity classification.

For each patient who received any statin therapy during their ICU stay, the modal daily dose (mg/day) was extracted from the MIMIC-IV prescription records and categorized according to the 2018 ACC/AHA cholesterol management guideline as low intensity (level 1, e.g., simvastatin 10 mg), moderate intensity (level 2, e.g., atorvastatin 20 mg), high intensity (level 3, e.g., atorvastatin ≥ 40 mg or rosuvastatin ≥ 20 mg) or “unclassified” (level 4, when the recorded dose could not be mapped to these categories) [16]. Statin intensity classification is shown in S1 Table. Because the primary research question focused on the effectiveness of statin therapy as a binary exposure (prescribed vs. not prescribed), statin intensity was not included in the primary model. Instead, it was used exclusively in a prespecified sensitivity analysis to evaluate the robustness of the primary outcomes.

### Covariates

The following variables were considered in this study: demographic characteristics (age, sex, race) and a broad range of comorbidities. These included cardiovascular conditions (e.g., myocardial infarction, peripheral vascular disease), neurological and cerebrovascular disorders (such as dementia and stroke), chronic respiratory and autoimmune diseases, diabetes (with or without complications), hepatic dysfunction across different severity levels, renal impairment, cancer (including metastatic tumors), sepsis, acquired immune deficiency syndrome(AIDS), and paraplegia. In addition to clinical diagnoses, we extracted laboratory measurements—such as platelet count, electrolyte levels (bicarbonate, calcium, sodium, potassium) and coagulation markers (INR). Vital signs and physiological parameters were also collected, including heart rate, blood pressure, respiratory rate, temperature and oxygen saturation. Furthermore, information on the types of statins administered during hospitalization was recorded. Due to the lack of structured echocardiographic data in the MIMIC-IV database, we were unable to include left ventricular ejection fraction (LVEF) as a variable in our analysis. Although LVEF is an important clinical indicator for heart failure phenotyping, it is primarily recorded as free text in echocardiography reports, rendering it unsuitable for large-scale quantitative analysis. As a result, we could not stratify patients by heart failure subtype (e.g., HFrEF vs. HFpEF), which constitutes a limitation of this study.

### Endpoints

We defined the primary endpoints as death due to any cause occurring within 90 and 180 days of admission. These outcomes were identified through hospital discharge records and follow-up information as recorded in the patients and admissions tables of the MIMIC database.

### Statistical adjustment and matching strategy

To control for potential confounding factors between groups, we conducted PSM [17,18] combined with Cox proportional hazards modeling. Matching was performed using a nearest-neighbor approach in a 1:1 ratio, with a caliper threshold of 0.2 standard deviations. The effectiveness of covariate balance after matching was assessed using standardized mean differences (SMDs). Group-level differences were analyzed using SMDs in combination with paired t-tests and chi-square tests for continuous and categorical variables, respectively.

To generate propensity scores, we incorporated demographic variables, key vital signs, relevant laboratory markers and a wide range of comorbidities, including but not limited to cardiovascular, cerebrovascular, respiratory, hepatic, renal and oncologic conditions.

Propensity score–based weights were applied to control for potential baseline imbalances. IPTW [19] and the SMRW method were employed to construct weighted cohorts that adjusted for baseline confounding, thereby improving the accuracy of the estimated association between statin use and mortality.

### Statistical analysis

Participants were grouped by statin usage for descriptive analysis. Statistical comparisons included parametric or nonparametric testing for continuous variables, and chi-square testing for categorical ones. Data distributions guided the reporting format—means ± SD for normal distributions and medians with IQR for skewed ones. The potential effect of statin therapy on survival outcomes was subsequently modeled using Cox proportional hazards regression. Additionally, we used the extended Cox model method for modeling with various covariate adjustments. Four sequential Cox regression models were constructed with progressive covariate adjustment. Model 1 included basic demographic variables (age, sex and race). Model 2 added key vital signs (heart rate, blood pressure, respiratory rate, body temperature and oxygen saturation). Model 3 incorporated laboratory parameters (platelet count, bicarbonate, calcium, sodium, potassium and INR). Model 4 further adjusted for a comprehensive range of comorbidities, including cardiovascular, neurological, respiratory, metabolic, hepatic, renal and oncological conditions, as well as AIDS and sepsis. A secondary Cox model was fitted with four-level statin intensity (level1–4) versus no statin. Furthermore, the Kaplan-Meier (KM) approach was utilized to plot survival curves, which were then subjected to log-rank testing. Subgroup analyses were performed for the covariates, including age, sex, heart rate, blood pressure, diabetes status, kidney disease status and sepsis status. Continuous variables such as age, heart rate and blood pressure were transformed into categorical variables based on clinical cutoff points.

To investigate whether survival benefits varied among specific statin subtypes, we further stratified statin-treated patients based on the most commonly prescribed agents in the database: atorvastatin, rosuvastatin, simvastatin and pitavastatin. Patients prescribed lovastatin were excluded from this exploratory analysis owing to limited sample size. Kaplan–Meier survival curves for 90-day and 180-day all-cause mortality were generated without covariate adjustment and visually compared among these statin subtypes, as the analysis was exploratory and aimed at hypothesis generation.

Statistical evaluations were performed with R version 3.6.3 (available at http://www.R-project.org, by The R Foundation) and Free Statistical software V.1.91. p < 0.05 (two-tailed) was considered to indicate statistical significance.

## Results

### Patient characteristics and outcomes

**Fig 1:**
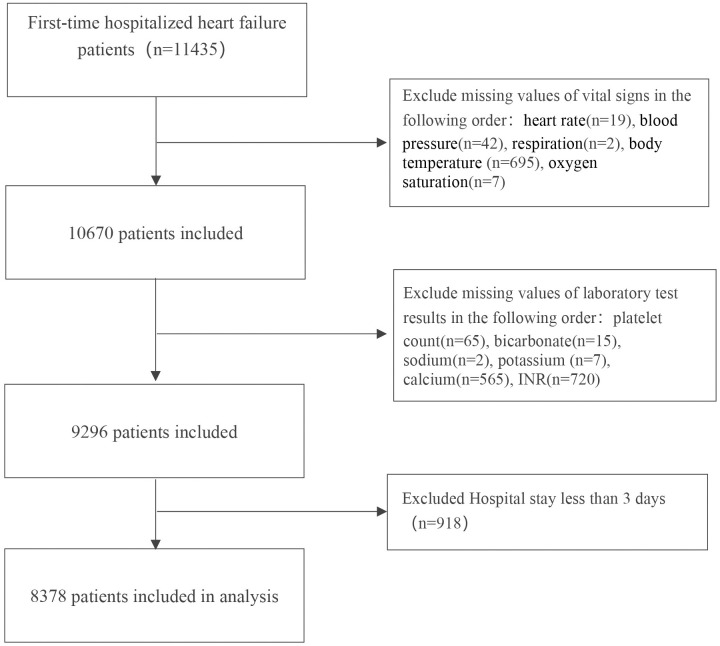
Flowchart of patient selection.

In this study, a total of 11,435 patients with heart failure caused by diverse reasons were retrieved from the database and following the inclusion and exclusion criteria, 8,378 patients ultimately participated in the analysis. The flowchart of the study is presented in Fig 1. In this cohort, 5,572 individuals (66.5%) were identified as statin users. The demographic and clinical features at baseline are presented in Table 1. The cohort had a mean age of 74.1 ± 13.4 years, with women accounting for 45.0% of the study population (n = 3,768). This study revealed that statin users tended to be elderly males and were more likely to have comorbidities, including myocardial infarction, diabetes, hypertension, cerebrovascular disease, renal disease and mild liver disease. It is important to note that statins are generally not prescribed for patients with severe liver disease, which is why the rate of statin use among these patients is significantly lower (165 (5.9) and 89 (1.6), p < 0.001). Except for statistically significant differences in calcium levels, platelet counts and the INR (p < 0.05), no notable differences were detected among the remaining laboratory test results. In this study, statin therapy was not significantly associated with the length of hospital stay (p > 0.05).

**Table 1:** The baseline characteristics of the study’s participants.

Variables	Total (n = 8378)	No Statin Use (n = 2806)	Statin Use (n = 5572)	p
Sex, N (%)				< 0.001***
Male	4610 (55.0)	1391 (49.6)	3219 (57.8)	
Female	3768 (45.0)	1415 (50.4)	2353 (42.2)	
Age (years)	74.1 ± 13.4	71.8 ± 16.0	75.3 ± 11.7	< 0.001***
Race (%)				< 0.001***
White	5863 (70.0)	1871 (66.7)	3992 (71.6)	
Black	790 (9.4)	296 (10.5)	494 (8.9)	
Asian	188 (2.2)	80 (2.9)	108 (1.9)	
Others	1537 (18.3)	559 (19.9)	978 (17.6)	
Heart rate (bpm)	103.1 ± 22.2	106.7 ± 23.1	101.3 ± 21.5	< 0.001***
SBP^a^ (mmHg)	146.4 ± 23.6	145.1 ± 23.8	147.1 ± 23.4	< 0.001***
DBP^b^ (mmHg)	88.3 ± 20.9	89.4 ± 21.1	87.7 ± 20.8	< 0.001***
Respiratory Rate (bpm)	29.0 ± 6.4	29.5 ± 6.6	28.8 ± 6.3	< 0.001***
Temperature (°C)	37.3 ± 0.7	37.3 ± 0.7	37.3 ± 0.7	0.088
Spo_2_ (%)	99.4 ± 1.1	99.4 ± 1.2	99.4 ± 1.1	0.446
Bicarbonate (mmol/L)	25.4 ± 4.8	25.4 ± 5.3	25.4 ± 4.6	0.495
Calcium (mmol/L)	8.7 ± 1.1	8.7 ± 0.8	8.7 ± 1.2	0.019
Sodium(mmol/L)	139.6 ± 5.0	139.6 ± 5.5	139.6 ± 4.7	0.911
Potassium (mmol/L)	4.7 ± 0.9	4.7 ± 0.9	4.7 ± 0.9	0.78
INR, Median (IQR)	1.3 (1.2, 1.7)	1.4 (1.2, 1.9)	1.3 (1.2, 1.7)	< 0.001***
Platelets (×10^9^/L)	210.0 (157.0, 278.0)	209.0 (150.0, 282.0)	210.0 (160.0, 276.0)	0.019
Myocardial Infarct	2763 (33.0)	379 (13.5)	2384 (42.8)	< 0.001***
Peripheral Vascular Disease	1413 (16.9)	308 (11)	1105 (19.8)	< 0.001***
Dementia	410 (4.9)	155 (5.5)	255 (4.6)	0.058
Cerebrovascular Disease	1189 (14.2)	314 (11.2)	875 (15.7)	< 0.001***
Chronic Pulmonary Disease	3169 (37.8)	1053 (37.5)	2116 (38)	0.689
Rheumatic Disease	372 (4.4)	136 (4.8)	236 (4.2)	0.2
Peptic Ulcer Disease	225 (2.7)	88 (3.1)	137 (2.5)	0.07
Diabetes	3460 (41.3)	824 (29.4)	2636 (47.3)	< 0.001***
Mild Liver Disease	774 (9.2)	427 (15.2)	347 (6.2)	< 0.001***
Paraplegia	333 (4.0)	89 (3.2)	244 (4.4)	0.008**
Renal Disease	3315 (39.6)	898 (32)	2417 (43.4)	< 0.001***
Malignant Cancer	912 (10.9)	385 (13.7)	527 (9.5)	< 0.001***
Severe Liver Disease	254 (3.0)	165 (5.9)	89 (1.6)	< 0.001***
Metastatic Solid Tumor	349 (4.2)	164 (5.8)	185 (3.3)	< 0.001***
AIDS^c^	21 (0.3)	14 (0.5)	7 (0.1)	0.001***
Charlson Comorbidity Index	7.6 ± 2.5	7.0 ± 2.7	7.9 ± 2.3	< 0.001***
Sepsis3	4585 (54.7)	1695 (60.4)	2890 (51.9)	< 0.001***
Length of stay(days)	8.8 (5.8, 14.0)	8.9 (5.9, 14.5)	8.8 (5.8, 13.9)	0.263
Hospital death	1015 (12.1)	451 (16.1)	564 (10.1)	< 0.001***
90-day mortality	2109(25.2)	863(30.8)	1246(22.4)	< 0.001***
180-day mortality	2537(30.3)	1002(35.7)	1535(27.5)	< 0.001***

*p < 0.05, **p < 0.01, ***p < 0.001

^a^SBP: SystolicBlood Pressure;

^b^DBP: Diastolic Blood Pressure;

^c^AIDS: Acquired Immune Deficiency Syndrome

### Results after Propensity Score Matching (PSM)

PSM was implemented to create 2,320 well-balanced matched pairs using a 1:1 algorithm. Covariate balance was confirmed through SMDs and visual inspection of propensity score overlap, with no notable group differences remaining (Table 2).

**Table 2:** Demographics and baseline characteristics of patients before and after propensity score matching.

Variables	Unmatched Patients			Propensity Score Matched Patients		
	No statin use	statin use	SMD	No statin use	statin use	SMD
n	2806	5572		2320	2320	
Female, sex, no. (%)	1415 (50.4)	2353 (42.2)	0.165	1139 (49.1)	1137 (49.0)	0.002
Age (years)	71.81 (16.05)	75.29 (11.68)	0.248	74.51 (14.16)	74.16 (12.90)	0.026
Race (%)			0.114			0.044
White	1871 (66.7)	3992 (71.6)		1603 (69.1)	1557 (67.1)	
Black	296 (10.5)	494 (8.9)		230 (9.9)	250 (10.8)	
Asian	80 (2.9)	108 (1.9)		61 (2.6)	66 (2.8)	
Others	559 (19.9)	978 (17.6)		426 (18.4)	447 (19.3)	
Heart rate (bpm)	106.67 (23.11)	101.34 (21.48)	0.239	104.55 (22.28)	104.48 (22.39)	0.003
SBP^a^ (mmHg)	145.05 (23.83)	147.13 (23.40)	0.088	146.30 (23.94)	146.42 (23.27)	0.005
DBP^b^ (mmHg)	89.40 (21.15)	87.71 (20.81)	0.081	88.90 (20.98)	88.63 (20.53)	0.013
Respiratory Rate (bpm)	29.54 (6.56)	28.75 (6.28)	0.123	29.20 (6.33)	29.22 (6.62)	0.003
Temperature (°C)	37.29 (0.74)	37.26 (0.65)	0.039	37.26 (0.71)	37.28 (0.69)	0.04
Spo_2_ (%)	99.41 (1.23)	99.43 (1.09)	0.017	99.41 (1.23)	99.42 (1.13)	0.013
Platelets (×10^9^/L)	227.45 (120.68)	229.51 (103.56)	0.018	231.03 (120.94)	229.40 (105.15)	0.014
Bicarbonate (mmol/L)	25.44 (5.31)	25.36 (4.57)	0.015	25.64 (5.36)	25.60 (4.67)	0.008
Calcium (mmol/L)	8.65 (0.81)	8.71 (1.20)	0.058	8.67 (0.79)	8.67 (0.72)	0.001
Sodium(mmol/L)	139.62 (5.49)	139.60 (4.66)	0.003	139.62 (5.31)	139.73 (4.93)	0.022
Potassium (mmol/L)	4.69 (0.92)	4.69 (0.85)	0.006	4.69 (0.92)	4.69 (0.88)	0.006
INR (Mean (SD))	1.93 (1.82)	1.70 (1.17)	0.153	1.81 (1.47)	1.84 (1.40)	0.022
Myocardial Infarct	379 (13.5)	2384 (42.8)	0.689	377 (16.2)	422 (18.2)	0.051
Peripheral Vascular Disease	308 (11.0)	1105 (19.8)	0.247	289 (12.5)	284 (12.2)	0.007
Dementia	155 (5.5)	255 (4.6)	0.043	134 (5.8)	135 (5.8)	0.002
Cerebrovascular Disease	314 (11.2)	875 (15.7)	0.133	285 (12.3)	311 (13.4)	0.033
Chronic Pulmonary Disease	1053 (37.5)	2116 (38.0)	0.009	889 (38.3)	871 (37.5)	0.016
Rheumatic Disease	136 (4.8)	236 (4.2)	0.029	105 (4.5)	103 (4.4)	0.004
Peptic Ulcer Disease	88 (3.1)	137 (2.5)	0.041	68 (2.9)	59 (2.5)	0.024
Diabetes	824 (29.4)	2636 (47.3)	0.375	776 (33.4)	794 (34.2)	0.016
Mild Liver Disease	427 (15.2)	347 (6.2)	0.294	231 (10.0)	238 (10.3)	0.01
Paraplegia	89 (3.2)	244 (4.4)	0.063	80 (3.4)	91 (3.9)	0.025
Renal Disease	898 (32.0)	2417 (43.4)	0.236	807 (34.8)	813 (35.0)	0.005
Malignant Cancer	385 (13.7)	527 (9.5)	0.133	280 (12.1)	293 (12.6)	0.017
Severe Live Disease	165 (5.9)	89 (1.6)	0.227	71 (3.1)	72 (3.1)	0.002
Metastatic Solid Tumor	164 (5.8)	185 (3.3)	0.121	116 (5.0)	121 (5.2)	0.01
AIDS^c^	14 (0.5)	7 (0.1)	0.067	6 (0.3)	6 (0.3)	<0.001
Charlson Comorbidity Index	7.03 (2.70)	7.91 (2.33)	0.346	7.24 (2.55)	7.28 (2.44)	0.016
Sepsis3	1695 (60.4)	2890 (51.9)	0.173	1337 (57.6)	1357 (58.5)	0.017

^a^SBP: Systolic Blood Pressure;

^b^DBP: Diastolic Blood Pressure;

^c^AIDS: Acquired Immune Deficiency Syndrome

### Association between statin therapy and mortality outcomes

As illustrated in the Table 3, analysis using an extended multivariable Cox model confirmed a consistent and statistically significant decline in mortality risk associated with statin therapy across both unadjusted and fully adjusted models (p < 0.001, Table 3). In the fully adjusted model (Model 4, Table 3), statin use was associated with a 37% lower risk of 90-day mortality (HR = 0.70, 95% CI:0.64–0.77, p < 0.001) and a 35% reduction in 180-day mortality risk (HR = 0.72, 95% CI:0.66–0.78, p < 0.001). Propensity score–adjusted analysis revealed a statistically significant decrease in both 90-day and 180-day mortality among patients treated with statins (HR = 0.72, 95% CI:0.64–0.80, p < 0.001; and HR = 0.72, 95% CI:0.65–0.80, p < 0.001, respectively; Table 3). IPTW further supported these results, yielding hazard ratios of 0.67 (95% CI:0.62–0.73, p < 0.001) and 0.68 (95% CI:0.63–0.74, p < 0.001), respectively. Likewise, standardized mortality ratio weighting (SMRW) produced consistent estimates.

**Table 3:** Association between statin use and mortality using a multivariate model approach and propensity-score analyses.

Variables	90-day mortality			180-day mortality		
	HR of statin use	95% CI	P value	HR of statin use	95% CI	P value
Crude model	0.68	0.62-0.74	<0.001*	0.72	0.66-0.77	<0.001*
Model 1	0.62	0.57-0.68	<0.001*	0.65	0.60-0.71	<0.001*
Model 2	0.66	0.60-0.72	<0.001*	0.69	0.63-0.74	<0.001*
Model 3	0.66	0.61-0.72	<0.001*	0.69	0.64-0.75	<0.001*
Model 4	0.70	0.64-0.77	<0.001*	0.72	0.66-0.78	<0.001*
Propensity Score adjusted	0.70	0.64-0.77	<0.001*	0.71	0.65-0.78	<0.001*
Propensity Score Matched	0.72	0.64-0.80	<0.001*	0.72	0.65-0.80	<0.001*
IPTW^a^	0.67	0.62-0.73	<0.001*	0.68	0.63-0.74	<0.001*
SMRW^b^	0.62	0.57-0.68	<0.001*	0.64	0.59-0.69	<0.001*

*p < 0.001

^a^IPTW: Inverse Probability Of Treatment Weighting

^b^SMRW: Standardized Mortality Ratio Weighting

**Fig 2:**
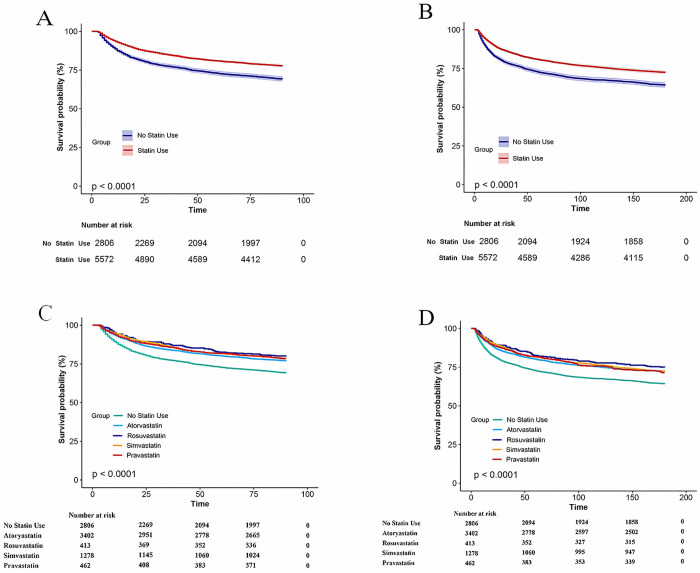
Kaplan-Meier survival curves. (A) Kaplan–Meier curves for 90-day all-cause mortality comparing statin users with non-users in the overall cohort (p < 0.0001). (B) Corresponding Kaplan–Meier curves for 180-day all-cause mortality (p < 0.0001). (C) Exploratory subtype-specific Kaplan–Meier analysis for 90-day mortality: atorvastatin, rosuvastatin, simvastatin and pitavastatin curves overlap; no significant between-subtype differences were observed. (D) Subtype-specific Kaplan–Meier analysis for 180-day mortality shows similar overlap among the four statin subtypes. Patients receiving lovastatin were excluded from the subtype analyses owing to limited sample size.

Furthermore, statin users—including those on different statin types—exhibited notably lower mortality at both 90 and 180 days compared to non-users (p < 0.0001) in the Kaplan–Meier survival plots (Fig 2). In exploratory analyses stratified by statin subtype, Kaplan–Meier survival curves for atorvastatin, rosuvastatin, simvastatin and pitavastatin showed largely overlapping trends for both 90-day and 180-day mortality. No major divergence in survival was observed among the subtypes. Patients prescribed lovastatin were excluded from this analysis due to limited sample size.

#### Sensitivity analysis by statin intensity.

Among 8,378 statin users, 384 (4.6%), 2,160 (25.8%), 2,868 (34.2%) and 160 (1.9%) were classified as low-intensity, moderate-intensity, high-intensity or unclassified-intensity users, respectively. As shown in S2 and S3 Tables, extended multivariable Cox models showed consistent, statistically significant reductions in mortality risk across statin intensity groups compared with no statin use (reference) in both unadjusted and fully adjusted analyses (p < 0.05). In the fully adjusted model, 90-day mortality was significantly lower among patients receiving low-intensity (HR 0.61, 95% CI 0.49–0.77, p < 0.001), moderate-intensity (HR 0.68, 95% CI 0.60–0.76, p < 0.001), high-intensity (HR 0.75, 95% CI 0.67–0.84, p < 0.001) and unclassified-intensity therapy (HR 0.68, 95% CI 0.48–0.97, p = 0.036). These associations remained consistent at 180 days: low-intensity (HR 0.67, 95% CI 0.55–0.82, p < 0.001), moderate-intensity (HR 0.69, 95% CI 0.62–0.77, p < 0.001), high-intensity (HR 0.75, 95% CI 0.68–0.84, p < 0.001) and unclassified-intensity (HR 0.73, 95% CI 0.53–1.00, p = 0.049). All estimates remained statistically significant.

### Stratified analyses

**Fig 3:**
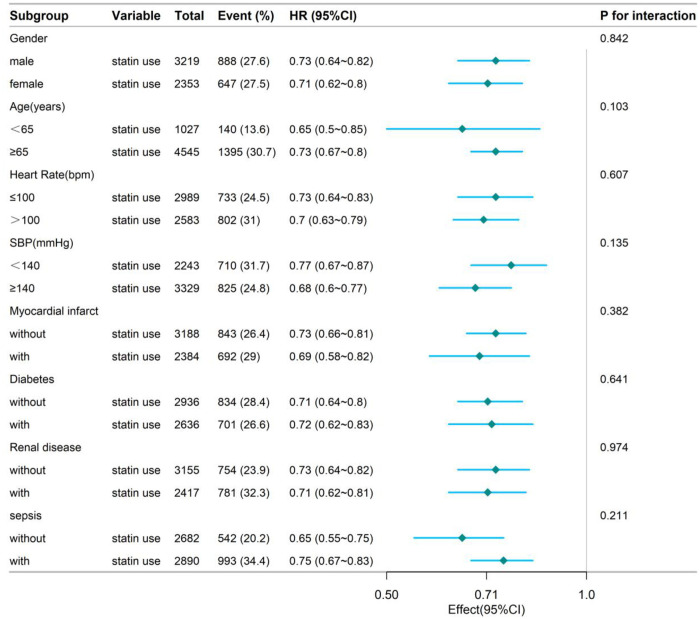
Stratified analyses by potential modifiers of the association between statin use and the risk of 90-day mortality.

**Fig 4:**
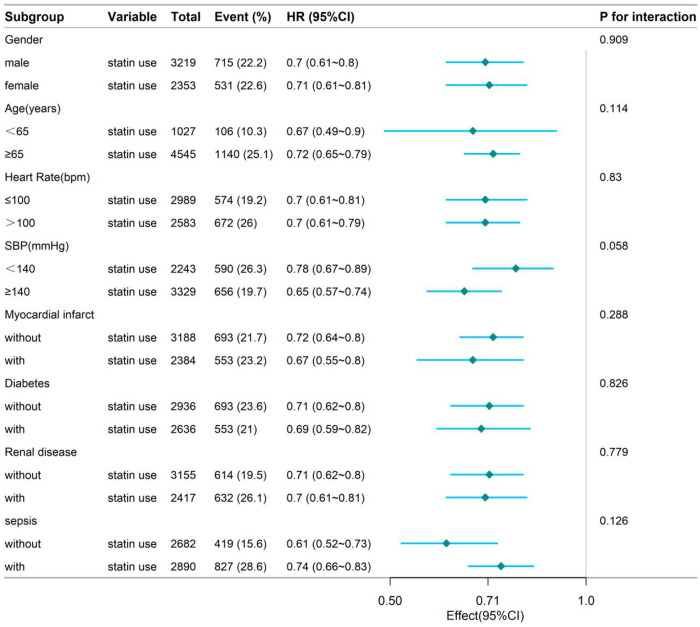
Stratified analyses by potential modifiers of the association between statin use and the risk of 180-day mortality.

To further explore the robustness of the observed association between statin therapy and mortality, we performed stratified and interaction analyses across clinically relevant subgroups, including age categories, sex, vital sign abnormalities and the presence of key comorbidities such as diabetes and sepsis. Remarkably, in-hospital statin use consistently demonstrated a protective effect on mortality across all subpopulations evaluated. As illustrated in Figs 3 and 4, the hazard ratios remained significantly below 1.0 within each stratum and no statistically significant interaction was detected (*P* for interaction > 0.05 for all variables). These findings highlight the broad applicability and potential clinical utility of statin therapy in diverse critically ill populations.

Forest plots display hazard ratios (HRs) with 95% confidence intervals (CIs) for the association between statin use and in-hospital mortality. Subgroups were defined a priori: age (<65 vs ≥ 65 years), sex (male vs female), abnormal vital signs (yes vs no), diabetes (present vs absent) and sepsis (present vs absent). The vertical dashed line indicates HR = 1.0 (no effect). All point estimates lie to the left of the null, with 95% CIs excluding 1.0, indicating a consistent protective effect of statins across subgroups. Interaction P-values for each covariate were >0.05, suggesting no statistically significant effect modification.

## Discussion

In this large-scale real-world study, the use of statins during hospitalization was significantly associated with reduced 90-day and 180-day all-cause mortality in critically ill heart failure patients, consistent with previous reports of similar benefits of statin therapy in heart failure patients [10,11,20,21]. Unlike earlier studies focusing on short-term outcomes (such as hospital or 28-day mortality), this study extends prior research by demonstrating an association between statin use during hospitalization and mid-term survival improvement (e.g., 90–180 days) in critically ill heart failure patients. While recent studies have reported short-term mortality benefits of statin therapy in ICU cohorts [22], our study provides additional evidence by reducing confounding factors and enhancing the robustness of the findings through the use of various causal inference techniques, such as propensity score matching (PSM) and inverse probability of treatment weighting (IPTW). To examine the robustness of the primary findings, we further stratified “statin use” into four groups based on prescription intensity (low, medium, high and unclassified) and refitted fully adjusted Cox models. The results showed that 90-day mortality was significantly lower across all intensity subgroups compared to the non-user group (HR range 0.61–0.75); 180-day mortality showed similar results (HR range 0.67–0.75). This sensitivity analysis further supports the study’s main conclusion, suggesting that in-hospital statin use may be associated with reduced 90-day and 180-day all-cause mortality among critically ill heart failure patients.

The clinical benefits of statins are well-established and widely accepted for both primary and secondary prevention of cardiovascular diseases [23,24]. In recent years, growing attention has been directed toward the pleiotropic properties of statins—effects that go beyond lipid lowering. Evidence indicates that statins may exert cardioprotective effects in the pathophysiological process of heart failure through diverse mechanisms, including suppression of systemic inflammation, stabilization of atherosclerotic plaques, enhancement of endothelial function, attenuation of sympathetic hyperactivation and reduction of oxidative stress [25–27]. Among these, their anti-inflammatory effects—mediated by downregulation of inflammatory mediators such as C-reactive protein (CRP), tumor necrosis factor-alpha (TNF-α) and interleukin-6 (IL-6)—help mitigate the inflammatory milieu associated with heart failure [28]. Plaque stabilization may reduce the risk of acute coronary events and thereby indirectly enhance left ventricular ejection fraction. Improvement in endothelial function increases vascular compliance and facilitates peripheral perfusion [29]. Moreover, statins appear to regulate autonomic nervous activity and reduce cardiomyocyte apoptosis and myocardial fibrosis [30]. Notably, these mechanisms are independent of cholesterol-lowering effects, highlighting the potential role of statins in heart failure beyond traditional lipid modulation pathways.

However, major Randomized controlled trials (RCTs)—including the CORONA and GISSI-HF trials—did not identify significant survival advantages associated with statin use in individuals with chronic heart failure, especially in those with reduced left ventricular ejection fraction. It is worth noting that RCTs have certain limitations, including narrow patient selection, stringent inclusion criteria and reliance on a single pharmacological intervention—all of which may restrict the generalizability of their findings to the broader and more heterogeneous real-world population [31,32]. Similarly, as demonstrated by the RCT-DUPLICATE demonstration project, discrepancies between real-world evidence and RCT results may stem from limited comparability in study populations, exclusion criteria and treatment parameters, such as initiation of therapy during hospitalization or discontinuation of baseline medications. A notable strength of this study lies in its use of a triangulation framework that integrates multiple analytic approaches to emulate key elements of RCT design and adjust for potential sources of bias and divergence [33]. By integrating a spectrum of confounding adjustment techniques rooted in distinct causal inference paradigms, this approach effectively emulated the counterfactual conditions of randomization within observational data. This design mitigated critical limitations of earlier trials—such as restricted generalizability and statin-specific constraints—thereby substantially enhancing internal validity. The consistent findings across PSM, IPTW and SMRW lend credibility to the observed associations, approximating the rigor of randomized trial evidence. Sensitivity analyses further indicated that variations in model specifications and covariate inclusion did not meaningfully alter the primary effect estimates, reinforcing the robustness of the conclusions.

We also performed the stratified Kaplan–Meier survival analyses by statin subtype in this high-risk population, focusing particularly on atorvastatin, rosuvastatin, simvastatin and pitavastatin. Although these agents differ pharmacologically—in terms of lipophilicity, potency and metabolic pathways—the survival curves largely overlapped. Lovastatin was excluded due to insufficient sample size, ensuring reliable subgroup comparisons and reflecting our emphasis on methodological rigor and data integrity.

Building upon these analyses, the findings open new avenues for investigating whether personalized statin selection based on pharmacokinetic characteristics or drug interaction profiles could further improve outcomes in critically ill patients with heart failure. Future prospective studies and mechanistic investigations are warranted to confirm these associations and elucidate underlying mechanisms.

Beyond the extended time horizon, the study also conducted detailed subgroup and interaction analyses to assess treatment effect heterogeneity across age, sex, vital signs, laboratory parameters and major comorbidities. The forest plots revealed consistent protective trends across all subgroups, without significant interactions. These findings suggest that the in-hospital benefits of statin therapy may be generalizable to a highly heterogeneous population with heart failure, thereby providing evidence to support individualized therapeutic strategies.

## Limitations

This study was based on a large-scale real-world database that included a wide range of heart failure patients, thereby more closely reflecting real-world clinical practice. However, several limitations should be noted. First, MIMIC-IV does not contain systematic echocardiographic data; quantitative LVEF values are missing for the majority of patients. Consequently, we were unable to classify admissions into HFrEF, HFmrEF, or HFpEF subgroups. This limitation precludes phenotype-specific effect estimates and may mask heterogeneous treatment responses across heart-failure phenotypes. Similarly, detailed NYHA functional class data are not recorded, preventing analyses of whether baseline symptom severity modifies statin efficacy. Second, even with rigorous adjustment strategies such as PSM and IPTW, the study’s retrospective design imposes inherent constraints on causal inference and the presence of residual confounding cannot be entirely excluded. Finally, information on statin type, dosage and treatment duration was limited, making it difficult to explore the impact of different treatment strategies on prognosis. Future studies should prospectively validate these findings in specific heart failure subtypes and investigate the effectiveness of different statin regimens to better inform personalized treatment.

## Conclusions

Taken together, this study provides compelling real-world evidence suggesting that appropriate in-hospital use of statins may confer short- and intermediate-term survival benefits in patients with heart failure. These findings merit further validation through prospective, multicenter studies featuring detailed phenotypic analyses and extended follow-up durations.

## Supporting information

S1 Table Statin intensity classification according to 2018 ACC/AHA guidelines (daily dose, mg). (DOCX)

S2 Table Analysis of the association between statin use at different dose intensities and 90-mortality using a multivariate model approach. (DOCX)

S3 Table Analysis of the association between statin use at different dose intensities and 180-mortality using a multivariate model approach. (DOCX)
