# Supplementary material for: Statin therapy and mortality in critically ill heart failure patients: Insights from a triangulated real-world design study
Source: PLoS One. 2025 Oct 17;20(10):e0334822. doi: 10.1371/journal.pone.0334822 (PMC12533876; doi:10.1371/journal.pone.0334822)
Supplement: S1 Table — (DOCX) [file pone.0334822.s001.docx]

**S1_Table** Statin intensity classification according to 2018 ACC/AHA guidelines (daily dose, mg)

| Statin | Low-intensity (1) | Moderate-intensity (2) | High-intensity (3) | Unclassified* |
| --- | --- | --- | --- | --- |
| Atorvastatin | — | 10–20 mg | 40–80 mg | — |
| Fluvastatin | 20–40 mg | 40 mg twice daily or ER 80 mg | — | — |
| Lovastatin | 20 mg | 40–80 mg | — | — |
| Pitavastatin | — | 1–4 mg | — | — |
| Pravastatin | 10–20 mg | 40–80 mg | — | — |
| Rosuvastatin | — | 5–10 mg | 20–40 mg | — |
| Simvastatin | 10 mg | 20–40 mg | — | — |

* Unclassified: All doses not explicitly listed in the above table (e.g. atorvastatin 15mg, rosuvastatin 8mg, etc.) are coded as "Unclassified/Other".
