# Supplementary material for: Statin therapy and mortality in critically ill heart failure patients: Insights from a triangulated real-world design study
Source: PLoS One. 2025 Oct 17;20(10):e0334822. doi: 10.1371/journal.pone.0334822 (PMC12533876; doi:10.1371/journal.pone.0334822)
Supplement: S2 Table — (DOCX) [file pone.0334822.s002.docx]

**S2_Table** Analysis of the association between statin use at different dose intensities and 90-mortality using a multivariate model approach

| **Variable** | Crude model |  | **Model 1** |  | **Model 2** |  | **Model 3** |  | **Model 4** |  |
| --- | --- | --- | --- | --- | --- | --- | --- | --- | --- | --- |
|  | **HR(95%CI)** | **P-value** | **HR(95%CI)** | **P-value** | **HR(95%CI)** | **P-value** | **HR(95%CI)** | **P-value** | **HR(95%CI)** | **P-value** |
| Statin | 0.68(0.62~0.74) | <0.001 | 0.62 (0.57-0.68) | <0.001 | 0.66 (0.60-0.72) | <0.001 | 0.66 (0.61-0.72) | <0.001 | 0.70(0.64-0.77) | <0.001 |
| statin  intensity |  |  |  |  |  |  |  |  |  |  |
| **No statin use** | 1(Ref) |  | 1(Ref) |  | 1(Ref) |  | 1(Ref) |  | 1(Ref) |  |
| Low-intensity | 0.66 (0.53~0.82) | <0.001 | 0.55 (0.44~0.68) | <0.001 | 0.58 (0.46~0.73) | <0.001 | 0.59 (0.47~0.74) | <0.001 | 0.61 (0.49~0.77) | <0.001 |
| Moderate-intensity | 0.67 (0.6~0.75) | <0.001 | 0.59 (0.52~0.66) | <0.001 | 0.63 (0.56~0.7) | <0.001 | 0.64 (0.57~0.71) | <0.001 | 0.68 (0.6~0.76) | <0.001 |
| High-intensity | 0.7 (0.63~0.77) | <0.001 | 0.67 (0.6~0.74) | <0.001 | 0.69 (0.63~0.77) | <0.001 | 0.7 (0.63~0.77) | <0.001 | 0.75 (0.67~0.84) | <0.001 |
| Unclassified | 0.6 (0.42~0.85) | 0.004 | 0.55 (0.39~0.78) | 0.001 | 0.6 (0.42~0.86) | 0.005 | 0.61 (0.43~0.87) | 0.006 | 0.68 (0.48~0.97) | 0.036 |

Model 1: age, sex, and race.

Model 2: Model 1 + heart rate, blood pressure, respiratory rate, body temperature, and oxygen saturation.

Model 3: Model 2 + platelet count, bicarbonate, calcium, sodium, potassium, and INR

Model 4: Model 3 + myocardial infarct, peripheral vascular disease, dementia, cerebrovascular disease, chronic pulmonary disease, rheumatic disease, peptic ulcer disease, diabetes, mild liver disease, paraplegia, renal disease, malignant cancer, severe liver disease, metastatic solid tumor, aids, charlson comorbidity index, sepsis3
